# Computer Vision for Cattle Health and Welfare Monitoring: A Comprehensive Review of Methods, Applications, and Interdisciplinary Integration in Smart Agriculture

**DOI:** 10.3390/s26134271

**Published:** 2026-07-04

**Authors:** Md Nafiul Islam, J. Lannett Edwards, Robert Burns, Hairong Qi, Hao Gan

**Affiliations:** 1 Department of Biosystems Engineering and Soil Science, University of Tennessee, Knoxville, TN 37996, USA; mdnafiul.islam@ag.tamu.edu (M.N.I.); rburns@utk.edu (R.B.); 2 Department of Animal Science, University of Tennessee, Knoxville, TN 37996, USA; jedwards@utk.edu; 3 Department of Electrical Engineering and Computer Science, University of Tennessee, Knoxville, TN 37996, USA; hqi@utk.edu

**Keywords:** computer vision, precision livestock farming, smart agriculture, cattle health monitoring, deep learning, machine learning

## Abstract

**Highlights:**

**What are the main findings?**

- Computer vision technologies (e.g., YOLO, CNNs, transformers, and 3D imaging) have achieved high accuracy (>90% in many cases) for key cattle health monitoring tasks, including BCS, lameness, weight, estrus, breathing, and behavior detection.
- A clear evolution is evident from traditional image processing to deep learning and multimodal systems, enabling real-time, non-invasive, and scalable livestock monitoring.

**What are the implications of the main findings?**

- These technologies can significantly improve precision livestock farming by enabling early disease detection, reducing labor requirements, and supporting data-driven decision-making for farmers and veterinarians.
- Future adoption will depend on overcoming challenges such as environmental variability, data annotation requirements, and system costs, with strong potential for multimodal AI and edge-based deployment.

**Abstract:**

The global cattle industry is experiencing significant growth, requiring advanced methods for monitoring animal health and welfare to ensure productivity and sustainability. Traditional manual monitoring techniques are labor-intensive and often impractical for large-scale operations. This review provides a comprehensive analysis of existing and emerging computer vision tools applied to the monitoring of cattle health and welfare. By systematically examining studies across major databases, this paper addresses six key research questions focusing on (1) the issues addressed by computer vision technologies, (2) data acquisition systems, (3) implemented techniques and algorithms, (4) performance outcomes, (5) challenges faced, and (6) potential applications for underexplored health and welfare aspects in cattle farming. The findings show that computer vision technologies have significantly progressed in areas such as body condition score detection, lameness detection, weight estimation, estrus detection, monitoring of feeding and drinking behavior, breathing detection, and recognition of general behaviors. Despite the progress, challenges such as variability in environmental conditions, the need for large annotated datasets, and the high cost of advanced imaging equipment persist. The review emphasizes future research opportunities to address these challenges by focusing on disease-specific monitoring. This review aims to provide veterinarians, farmers, and animal health professionals with greater insight into computer vision technologies and to promote their adoption by discussing their practical applications.

## 1. Introduction

The global beef market was estimated at $436.60 billion in 2023 and is expected to expand from $459.87 billion in 2024 to $656.44 billion by 2032, reflecting a compound annual growth rate (CAGR) of 5.52% over the forecasted period [1]. Similarly, the dairy cattle market had a valuation of approximately $944.7 billion in 2023 and is projected to grow to $1459.3 billion by 2032, with a CAGR of 4.95% during the same period [2]. The cattle industry is experiencing significant growth; however, modern cattle farming faces considerable challenges in balancing productivity with animal health and welfare. Traditionally, monitoring cattle health depends on manual methods, which can be labor-intensive, prone to inaccuracies, and difficult to scale efficiently, especially in large-scale farming operations [3].

Recent advancements in computer vision technology have the potential to transform cattle farming by providing farmers with innovative tools to monitor key health and welfare indicators through non-invasive, automated systems [4]. By integrating cameras with sophisticated algorithms, these computer vision systems can continuously observe cattle, analyzing their behaviors, physical attributes, and overall well-being in real-time [5]. These systems have the capability to detect early warning signs of potential health issues. Unlike traditional methods that often require physical handling and can cause animal stress, automated solutions enable continuous monitoring in a more stress-free environment. Additionally, the data collected from these systems can enhance decision-making, improve herd management practices, and promote better animal welfare, all while reducing labor demands and increasing the scalability of monitoring efforts on large farms [6].

The rapid advancement of computer vision technologies in areas such as machine learning, deep learning, and 3D imaging has significantly improved the ability of researchers and professionals to monitor cattle health with remarkable accuracy [7]. Additionally, state-of-the-art computer vision applications utilize sophisticated models to detect subtle animal welfare indicators, such as early signs of stress, potential illnesses, and reproductive readiness, factors often challenging to spot through traditional observation methods. Providing actionable insights into cattle health and behavior, these systems help farmers proactively address issues and promote more sustainable livestock management practices [8].

Existing review papers have extensively examined the integration of precision livestock farming (PLF) technologies in sustainable cattle farming, with a strong focus on machine learning and deep learning applications [9,10,11,12,13,14]. These reviews highlighted the details of the machine learning and deep learning methods, particularly those that utilized sensors and camera systems, to improve various aspects of cattle farming, including health monitoring, cattle identification, grazing behavior analysis, and overall farm management efficiency. For instance, Lovarelli et al. (2020) [15] explored the role of PLF in addressing the increasing demand for sustainable dairy farming, while Mahmud et al. (2021) [7] provided an in-depth overview of deep learning applications in cattle health monitoring and identification.

Unlike existing reviews, this review examines specific cattle health and welfare problems and discusses how computer vision technologies can be applied to solve them. The review will explain innovative concepts and applications of computer vision for cattle farming, covering a wide range of models and use cases. Additionally, the review strives to minimize the use of technical terms and concepts in computer vision and present the topics to a broader audience, including animal health experts, veterinarians, and farmers. The goal is to help reduce barriers to the adoption of computer vision and other PLF technologies for cattle health and welfare monitoring, and to promote more effective management in cattle farming.

## 2. Materials and Methods

### 2.1. Research Questions

This review paper examines studies on the use of computer vision techniques in cattle farming. The selected studies were analyzed from different perspectives, leading to the development of the following six research questions (RQs):

RQ1: What specific issues in cattle farming have been addressed using computer vision technologies?

RQ2: What types of data acquisition systems have been used in computer vision applications for cattle farming?

RQ3: Which computer vision techniques and algorithms have been implemented in the context of cattle farming?

RQ4: Which computer vision techniques have demonstrated the highest performance in addressing specific challenges in cattle farming?

RQ5: What challenges are faced when applying computer vision techniques in cattle farming?

RQ6: How could underexplored health and welfare benefit from utilizing existing computer vision models in cattle farming?

### 2.2. Databases and Search Strategy

This review focuses on camera-based methods for monitoring cattle health and welfare using computer vision techniques. A systematic search strategy was designed to identify relevant studies published between January 2015 and May 2026, ensuring comprehensive coverage of recent advances in this area. The search was conducted across six major databases: ScienceDirect, Google Scholar, Scopus, Web of Science, Wiley, and SpringerLink. The initial search was framed using broad keywords to maximize the retrieval of relevant studies. The general search string included terms related to cattle, monitoring methods, and computer vision technologies, as follows:

Cattle Terms: “dairy cow” OR “beef cattle” OR “calf” OR “cattle” OR “cow” OR “heifer”

Monitoring Terms: “monitoring” OR “detection” OR “recognition” OR “tracking”

Technology Terms: “computer vision” OR “deep learning” OR “machine learning”

The combined search equation was:

(“dairy cow” OR “beef cattle” OR “calf” OR “cattle” OR “cow” OR “heifer”) AND 

(“monitoring” OR “detection” OR “recognition” OR “tracking”) AND 

(“computer vision” OR “deep learning” OR “machine learning”)

Each database’s search capabilities and constraints were considered during the query formulation process:

ScienceDirect: Due to the limit of eight Boolean (AND/OR) operators in advanced searches, the query was simplified by adjusting the cattle terms to (“cattle” OR “cow”), reducing the size of the search equation while retaining relevance.

Google Scholar: Google Scholar imposes a character limit on search queries. Consequently, the search was split into two strings, and the results from both queries were combined. Duplicate entries were removed during the data cleaning process.

Other Databases (Scopus, Web of Science, Wiley, SpringerLink): The full search string was used without modification, leveraging these platforms’ advanced search capabilities.

The search strategy aimed to capture studies that employed camera-based methods to monitor cattle health and welfare. Studies addressing dairy cows, beef cattle, calves, or heifers were included, provided they applied computer vision, deep learning, or machine learning techniques. Only papers published in English between January 2015 and May 2026 were considered. This comprehensive search strategy ensured the inclusion of a wide array of studies relevant to the review’s focus on advancements in computer vision applications for monitoring cattle health and welfare.

### 2.3. Selection Criteria

Specific inclusion and exclusion criteria were defined and applied systematically to ensure the inclusion of relevant and high-quality studies in this review. These criteria helped set boundaries for the review process and filtered out studies that did not align with the focus on computer vision applications for cattle health and welfare monitoring. The following exclusion criteria (EC) were applied:

EC.1: The publication does not relate to computer vision methods for cattle farming, including health and welfare monitoring.

EC.2: The publication is duplicated or retrieved from multiple databases. Duplicate studies were identified and removed.

EC.3: The publication is a survey or review paper rather than an original research study.

EC.4: The full text of the study is not available for review or analysis.

EC.5: The publication is not peer-reviewed, ensuring only credible, scholarly articles are included.

EC.6: The study is not published in the English language.

EC.7: The publication date is before January 2015, as the review focuses on recent advancements in computer vision techniques (January 2015 and May 2026).

**Figure 1:**
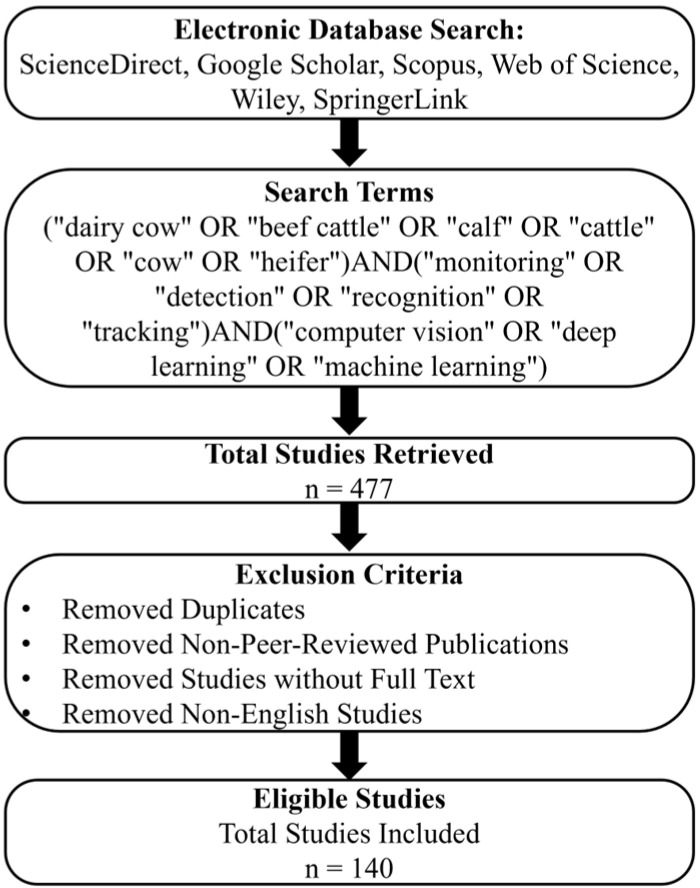
Flowchart of the literature search strategy and study selection procedure.

All duplicate studies were identified and removed. Titles and abstracts were screened to exclude ineligible papers based on EC.1. Remaining studies were assessed for eligibility, excluding non-peer-reviewed papers, survey/review articles, and studies unavailable in full-text or non-English publications. Only studies meeting all inclusion criteria were retained, resulting in a final set of 140 studies. The final set of 140 studies focused on various detection methods applied to monitor cattle health and welfare. These methods can be categorized into key areas based on their objectives and techniques. The topics identified include body condition score (BCS) estimation, lameness detection, weight estimation, estrus detection, monitoring of drinking and feeding behavior, and basic behavioral observations. Figure 1 shows the flowchart of the literature search strategy and study selection procedure. Table A1 also provides more details on the search strategies and the retrieved records.

## 3. Results

### 3.1. BCS Detection

An established method in cattle farming is to use BCS to indirectly assess the mobilization of energy reserves from adipose tissue and muscle. The systematic use of BCS is prevalent in monitoring feed intake and health status, serving as an analytical tool in optimizing both meat and milk production management [16,17]. The precise and consistent assessment of BCS demands certain expertise in visual and tactile techniques, relying on the observation of specific anatomical features such as the shape of the cow at the chine, loin, and rump, as well as the assessment of the ribs, spinous processes (specifically in the loin area), tuber sacral (hip or hook bones), tuber ischii (pin bones), anterior coccygeal vertebrae (tail head), and the thigh region [18]. However, this manual approach is both time-consuming and subjective, introducing the possibility of inaccuracies arising from bias. In response to these challenges, recent advancements in technology have sought to automate the BCS evaluation process. Electronic visual systems, employing various types of cameras, have been developed to analyze the contours of cows. Remarkably, these automated systems have demonstrated a robust correlation between the observations made manually and the technological representations, offering a promising avenue to enhance the accuracy and efficiency of BCS assessments [19,20,21,22,23].

Automated technologies have been developed using various computer vision and computational methods to increase the accuracy and effectiveness of BCS assessments. These technologies mainly fall into several categories: thermal imaging, cost-effective RGB camera systems that use Convolutional Neural Networks (CNNs), advanced three-dimensional imaging techniques, and integrated sensor systems incorporating ultrasound technology. Thermal imaging uses infrared technology to identify temperature differences linked to fat distribution in different body areas. Advanced 3D imaging technologies, including depth imaging and point cloud analyses, allow for accurately capturing and measuring the cow’s body shape. In addition, ultrasound sensors integrated within these systems provide direct measurements of fat and thickness. Together, these technological advancements significantly improve BCS evaluations’ precision, consistency, and practicality, offering promising, adaptable solutions for various cattle farming management scenarios. These methods have been widely validated, particularly in dairy cattle, where deep learning techniques consistently achieve accuracy rates typically exceeding 90%. The existing automatic BCS methods using computer vision are described below.

#### 3.1.1. Affordable RGB Imaging and Deep Learning Models

Affordable RGB imaging combined with deep learning has become one of the most widely adopted approaches for automated BCS estimation due to its cost-effectiveness and scalability. Yukun et al. (2019) [24] integrated manual BCS scores and ultrasound backfat measurements with convolutional neural network (CNN)-based models to improve prediction accuracy, incorporating attention mechanisms such as squeeze-and-excitation modules to enhance feature representation [24].

In the same year, Huang et al. (2019) [25] developed a low-cost monitoring system using network cameras and a Single Shot MultiBox Detector algorithm, focusing on key anatomical regions such as the tailhead and backbone to achieve high classification accuracy [25].

Subsequent advancements further improved model performance and efficiency. Wu et al. (2021) [26] compared traditional CNN architectures, including ResNet and EfficientNet, with transformer-based models such as the Swin Transformer, demonstrating that attention mechanisms significantly enhance prediction accuracy, achieving over 97% agreement with manual scoring within a 0.25-unit margin [26].

More recent studies emphasize lightweight and deployable models. Liu et al. (2025) [27] utilized EfficientNet-based architectures to enable real-time BCS classification on edge devices, while Zheng et al. (2024) [28] proposed a YOLO-based framework optimized through knowledge distillation for faster and more efficient inference [27,28]. Overall, these studies highlight the transition toward practical, non-intrusive, and scalable solutions, making automated BCS accessible to farms with limited resources.

#### 3.1.2. Advanced 3D Imaging and Shape Analysis

Advanced 3D imaging techniques have been introduced to overcome the limitations of 2D image-based methods by capturing the structural geometry of the cow’s body. These approaches utilize depth cameras and point cloud data to analyze surface concavity and fat distribution, which are critical indicators of body condition. Liu et al. (2020) [29] employed Gaussian Mixture Models for background segmentation and extracted 3D geometric features based on anatomical landmarks such as the spine, hook bones, and tailhead [29]. Similarly, Shi et al. (2023) [23] combined depth imaging with PointNet++ to process point cloud data and improve feature extraction from the posterior region of dairy cows [23]. Zhao et al. (2023) [30] further advanced this domain by introducing a shape analysis framework that quantitatively compares 3D body surfaces to derive BCS [30]. While these methods provide a more detailed representation of body structure and improve prediction accuracy, they often face challenges related to noise in point cloud data, computational complexity, and higher equipment costs. As a result, their application is more common in research settings than in large-scale commercial farms.

#### 3.1.3. Integrated Ultrasound and RGB Imaging Systems

Integrated systems combining ultrasound and RGB imaging have been explored to enhance the reliability of BCS estimation by incorporating both visual and physiological indicators. Ultrasound measurements provide direct quantification of backfat thickness, which is strongly correlated with body condition, while RGB imaging enables automated and non-invasive monitoring. Yukun et al. (2019) [24] demonstrated that combining ultrasound-derived backfat thickness with CNN-based image analysis improves model performance by providing complementary information for training [24]. Similarly, Tao et al. (2022) [31] utilized portable ultrasound devices alongside 3D imaging systems to validate automated BCS predictions, achieving strong agreement with manual scoring methods [31]. Although these multimodal approaches offer higher accuracy and robustness, their dependence on additional equipment and manual intervention limits their practicality for continuous monitoring. Consequently, they are primarily used for validation purposes or in controlled research environments rather than routine farm operations. Table 1 summarizes the cattle body condition score method using computer vision.

### 3.2. Lameness Detection

Lameness is a painful condition that affects the locomotor system of cattle, resulting in abnormal gait or movement [52]. It is a significant welfare and economic issue in both dairy and beef cattle industries, though it tends to receive more attention in dairy cattle. Lameness can be caused by various factors, with the majority (70–90%) of cases involving hoof lesions, which can be either infectious (e.g., digital dermatitis, foot rot) or non-infectious (e.g., sole ulcers, white line disease) [53,54,55]. Lameness affects the cow’s ability to walk normally, impacting feeding, milk production, reproductive performance, and overall quality of life [56]. Manual detection of lameness relies on visual locomotion scoring, commonly using a 5-point scale. Observers examine the cow’s movement, stance, and behavioral cues to assign a score ranging from 1 (normal gait) to 5 (severely lame) [57]. In practice, locomotion scoring involves evaluating specific behaviors that indicate discomfort. Cattle with lameness may exhibit an arched back while walking to alleviate pain, particularly when taking weight off an affected limb. Head bobbing is another common sign, as cows adjust their head movement to counterbalance the pain during movement [58]. Shortened or uneven strides, referred to as asymmetric gait, are also indicative of lameness, as cows attempt to minimize the pressure on painful areas [59]. Observers also assess stance and weight-bearing behaviors; lame cows often shift their weight or favor certain limbs, displaying a reluctance to fully bear weight on the affected side [60]. Behavioral changes, such as reduced speed, irregular walking patterns, or difficulty in rising, further indicate the severity of lameness [58]. While locomotion scoring provides valuable insights, manual detection has limitations. The process is time-consuming, labor-intensive, and subjective, as scoring accuracy can vary significantly based on the observer’s experience and interpretation [56]. Subtle signs of early lameness may go undetected without frequent monitoring, especially in large herds [61].

Various automated approaches employing computer vision technologies have been developed for accurate and timely lameness detection. These automated methods can be broadly grouped into several categories: traditional and depth-based video analysis, advanced object detection and localization techniques, pose estimation and tracking, and thermal imaging. Traditional and depth-based video analysis utilizes RGB or depth cameras to analyze stride patterns, hoof-ground contact times, and back curvature. Object detection and localization methods typically utilize deep learning algorithms, such as YOLO models, to detect and track specific body regions like the back, limbs, and hooves for curvature and movement analysis. Pose estimation and tracking methods integrate segmentation and tracking models like Mask-RCNN and YOLO, analyzing head movements, back posture, and overall gait patterns. Thermal imaging techniques identify temperature variations indicative of inflammation and joint issues associated with lameness. These methods collectively improve reliability, efficiency, and early detection of lameness across farming operations. Most studies have focused on dairy cows, with reported accuracy rates ranging from 71.9% to 98.89%. The existing methods of automatic lameness techniques using computer vision are described below.

#### 3.2.1. RGB, Depth, and Thermal Video Analysis

Multimodal imaging approaches combining RGB, depth, and thermal data have significantly enhanced the robustness of lameness detection systems. RGB and depth cameras are widely used to capture gait and posture, while thermal imaging provides additional physiological information by detecting inflammation-related temperature changes. For example, Coşkun et al. (2023) [62] utilized thermal imaging to identify temperature variations in the fetlock joint, achieving high accuracy in distinguishing lame and healthy cows.

More recent studies have integrated thermal imaging with deep learning for automated analysis. Bumbálek et al. (2026) [63] combined infrared thermography with YOLOv8-based detection and classification models to identify abnormal hoof temperatures associated with lameness, enabling early detection in a non-invasive manner [63]. These multimodal approaches improve detection reliability by capturing both visual and physiological indicators; however, they may require additional hardware and calibration, increasing system complexity.

#### 3.2.2. Object Detection and Localization Methods

Object detection and localization play a fundamental role in lameness detection by identifying key body parts such as hooves, back, and joints within images or video frames. Deep learning models, particularly those based on the YOLO and SSD architectures, have been widely used for this purpose. Kang et al. (2020) [64] applied an RFB_Net_SSD model to accurately detect hoof positions and calculate supporting phase durations, enabling the identification of uneven gait patterns associated with lameness. Similarly, Jia et al. (2023) [65] employed a lightweight YOLO-based model (GhostNet_YOLOv4) to detect the cow’s back region and analyze curvature changes during movement, achieving high detection accuracy.

More advanced systems combine detection with segmentation and keypoint estimation. Barney et al. (2023) [66] utilized Mask R-CNN to identify anatomical key points and derive posture-related features, while Myint et al. (2024) [67] integrated YOLOv8 and Mask R-CNN for detection and tracking in video streams. These methods provide precise localization of relevant body regions, forming the foundation for extracting meaningful features for lameness classification.

#### 3.2.3. Pose Estimation and Tracking

Pose estimation and tracking techniques have further advanced lameness detection by enabling detailed analysis of animal movement over time. These methods focus on tracking key body points such as the head, back, and hooves to quantify motion patterns and detect abnormalities. Zhao et al. (2023) [68] used DeepLabCut (v2.2b8) to track multiple body points and derive motion features such as stride asymmetry and movement speed, achieving high classification accuracy [68].

Recent studies have expanded this approach by integrating temporal modeling. Jia et al. (2025) [69] combined pose estimation with deep learning models, including convolutional neural networks and LSTM-based architectures, to analyze temporal changes in posture and gait [69]. Similarly, Narli et al. (2025) [70] utilized DeepLabCut to extract back posture features and applied deep neural networks to classify lameness based on curvature patterns [70].

These approaches provide a more comprehensive understanding of movement dynamics, enabling earlier and more accurate detection of lameness compared to static image-based methods.

#### 3.2.4. Temporal Gait Analysis and Motion Modeling

In addition to spatial feature extraction, temporal analysis of gait patterns has become increasingly important in lameness detection. Techniques such as History Energy Images (HEI) and Gait Energy Images (GEI) capture motion information over time, highlighting irregularities in movement. Li et al. (2024) [71] utilized these representations in combination with MobileNet-based classifiers to analyze gait patterns and improve detection accuracy.

Other approaches focus on measuring biomechanical features such as stride length, support phase duration, and body sway. Higaki et al. (2025) [72] extracted movement indicators from tracked body points and applied a Random Forest classifier to detect lameness, providing an interpretable framework for mobility assessment [72]. These temporal modeling techniques enhance robustness by capturing dynamic behavioral changes that are not visible in single-frame analysis. Table 2 summarizes the cattle lameness detection using computer vision.

### 3.3. Weight Estimation

Weight estimation in cattle is a crucial aspect of livestock management and is vital in various aspects of livestock farming. Accurate weight assessments enable farmers and ranchers to make informed decisions regarding feed management, health monitoring, breeding programs, and market readiness. Producers can optimize growth rates by regularly estimating or measuring cattle weights, detecting health issues early, administering correct medication doses, and determining the ideal time for market sale [81]. While electronic scales provide the most precise measurements, manual estimation techniques such as heart girth measurements, body length calculations, and visual assessments by experienced handlers can offer reasonably accurate approximations when scales are unavailable. Although less precise, these manual methods provide valuable data for day-to-day management decisions. Computer vision technology for cattle weight estimation is rapidly evolving, potentially significantly improving livestock management practices, particularly for smallholder farmers in developing regions [82].

Automated computer vision techniques for estimating cattle weight are primarily divided into two categories: 3D imaging methods, which involve depth cameras and point cloud analysis, and traditional machine learning approaches that use historical data. The 3D imaging techniques utilize depth-sensing devices and LiDAR sensors to gather accurate spatial data and physical shapes, allowing for precise weight estimations based on cattle dimensions, including length, height, width, and body curvature. In contrast, traditional machine learning methods depend on supervised regression models that are trained with historical data on cattle, considering factors like breed, age, and gender to forecast weight gain and determine ideal body weight. The majority of weight estimation studies focused on dairy cows, with reported accuracies ranging from 88% to 98%.

#### 3.3.1. 3D Imaging and Depth Cameras

3D imaging and depth cameras have evolved significantly over time to estimate cattle weight. Nir et al. (2018) [83] developed a system using a Microsoft Kinect V2 camera to capture depth images of heifers. The system detects body shapes, fits ellipses to approximate size, and extracts body length, width, and volume measurements to estimate weight. Later, Bezsonov et al. (2021) [84] enhanced this technique by incorporating depth map generation. Their approach used Mask R-CNN to detect contours in synchronized images captured from multiple angles, while stereopsis and epipolar geometry calculated depth maps. Key dimensions such as height, length, and hip width were then processed through an MLP model to predict weight. Lassen et al. (2023) [85] focused on analyzing back curvature using a 3D camera to capture the shape and curve of cows’ backs. By mapping 100 points along the spine, the system connected curvature data to weight estimation models. Similarly, Bi et al. (2023) [86] employed depth-sensing cameras with Mask R-CNN to isolate top-view images of cows, measuring features like length, width, height, and volume for weight predictions.

#### 3.3.2. 3D Point Cloud Data

The integration of 3D point cloud data has allowed for more detailed spatial analysis in weight estimation. Hou et al. (2023) [87] employed LiDAR sensors to capture high-resolution 3D point cloud data of cattle. Using the PointNet++ model, they extracted localized measurements such as body length and chest girth through advanced curvature analysis. These measurements, segmented into regions like the back, chest, and hip, were combined with Johnson’s formula to provide accurate weight predictions. Contour-based techniques have also gained prominence in cattle weight estimation. Gebreyesus et al. (2023) [88] utilized 3D contour data captured through cameras to create detailed models of cattle shapes. These contours were processed using various supervised learning models, including ridge regression, random forest, and AdaBoost, to predict weight accurately.

#### 3.3.3. Traditional Machine Learning

Traditional machine-learning approaches have focused on leveraging historical cattle data for weight estimation. Garcia et al. (2021) [89] applied supervised regression models such as DT, GB, KNN, and RF. These models utilized features like age, breed, and gender, achieving robust predictions of weight gain and ideal body weight using historical records. More recent work has integrated machine learning with computer vision-derived features. Ruchay et al. (2026) [90] developed a system that combines image-based body measurements and behavioral indicators, such as walking speed, captured using RGB and depth cameras. A YOLOv8-based detection model was used to track cattle, and extracted features were processed using an ExtraTreesRegressor to estimate body weight accurately [90].

Similarly, Liao et al. (2025) [91] utilized depth imaging combined with YOLOv8-based segmentation to extract body dimensions of calves, including length, width, height, and volume. These features were then used in predictive models such as Extreme Gradient Boosting (XGBoost), linear regression, and linear mixed models to estimate and track weight over time [91]. Table 3 summarizes the cattle weight estimation method using computer vision.

### 3.4. Estrus Detection

Inseminating a cow during the appropriate window of time (either by the bull or artificially) is critically important for reproductive success. Historically, farmers employed conventional methods to detect this fertile period, collectively known as estrus, by recognizing sexually based behaviors. Happening once every 17–24 days, cows exhibiting estrus not only stand to be mounted by others but also mount other cows and engage in other activities such as sniffing and chin-resting on fellow cows [92,93]. Ref. [93] The conventional monitoring of such behaviors is labor-intensive, expensive, and susceptible to errors. Using animal-based monitoring is more dependable and informative than conventional methods. In this approach, farmers frequently depend on behavioral cues and physiological markers to evaluate animal well-being. These physiological markers include body temperature, heart rate, respiratory rate, and lesion or injury identification [94]. Automated activity monitoring technologies are useful to identify an estrus event and provide an alternative to supplement or, in some production systems, replace conventional estrus detection [95].

Specific to computer vision methods for estrus detection, these primarily fall into two categories: behavioral analysis through machine learning models and physiological monitoring via thermal imaging techniques. Machine learning-based systems utilize advanced algorithms like YOLO variants and neural networks to accurately identify estrus-specific behaviors, including mounting and increased activity levels, in real-time. Thermal imaging approaches capitalize on temperature fluctuations associated with estrus, often using infrared cameras combined with supervised learning algorithms to detect physiological changes reliably.

#### 3.4.1. Machine Learning Models

Machine learning and deep learning approaches have become central to automated estrus detection by enabling the identification of behavioral patterns associated with reproductive cycles. Early studies primarily relied on motion analysis and background subtraction techniques. For instance, Guo et al. (2019) [96] utilized background subtraction and motion features to detect mounting behavior, achieving an accuracy of 90.9%. Similarly, Higaki et al. (2021) [97] monitored activity levels and identified increased movement during estrus periods using image-based motion detection methods.

Subsequent research incorporated more advanced computer vision and deep learning models to improve detection performance. Arago et al. (2020) [98] applied image processing combined with neural networks to identify standing heat behaviors, although with moderate efficiency. More robust approaches emerged with object detection frameworks; Wang et al. (2022) [99] employed YOLOv5 to detect mounting behavior, achieving 97% precision and 89.5% recall under complex farm conditions.

Recent advancements have focused on integrating multiple models and optimizing architectures for higher accuracy. Arıkan et al. (2023) [100] combined VGG-19 with YOLOv5 to improve mounting detection, achieving 94% accuracy, while Lodkaew et al. (2023) [101] integrated YOLOv4 with machine learning models such as XGBoost and CatBoost to analyze multiple behaviors, including mounting and sniffing, thereby enhancing prediction performance. In addition, Chae and Cho (2021) [102] improved YOLOv3 with advanced activation functions, achieving 98% precision and 97% recall for real-time estrus detection.

More recent studies have further expanded behavioral analysis using spatiotemporal modeling. Ninphet et al. (2024) [103] employed convolutional neural networks combined with YOLOv5 to classify behaviors such as walking, mounting, and mating from CCTV footage, improving automated estrus prediction. Aryawan et al. (2024) [104] focused on posture-based activity patterns, using YOLOv5 and pose estimation models to quantify standing and lying behaviors, which are key indicators of estrus.

Additionally, multi-view and temporal tracking approaches have improved detection robustness. Hanpinitsak et al. (2026) [105] integrated multi-angle video analysis using YOLOv8 and ensemble fusion techniques to accurately detect mounting and chin-resting behaviors. Similarly, Wang et al. (2025) [106] combined YOLOv5 with DeepSORT tracking and signal processing techniques such as Fast Fourier Transform (FFT) and Principal Component Analysis (PCA) to analyze movement patterns over time, enabling more reliable estrus detection in dynamic environments. Overall, these studies demonstrate a clear progression from simple motion-based methods to advanced deep learning frameworks capable of capturing complex behavioral and temporal patterns, significantly improving the accuracy and scalability of estrus detection systems.

#### 3.4.2. Thermal Imaging

Thermal imaging has been increasingly utilized in estrus detection due to its ability to capture physiological changes associated with reproductive cycles. During estrus, cows exhibit temperature variations in specific body regions, particularly around the vulva and neck, which can serve as reliable indicators. Perez Marquez et al. (2022) [107] combined infrared thermal imaging of vulva temperature with tail movement tracking to detect estrus events, demonstrating the effectiveness of integrating physiological and behavioral signals.

Similarly, Wang et al. (2023) [108] applied thermal infrared imaging combined with machine learning models such as Support Vector Machines (SVM) to classify estrus based on temperature fluctuations. These approaches provide a non-invasive alternative to traditional methods and reduce reliance on manual observation.

In addition to thermal imaging, behavioral and physiological data integration has shown promising results. Cairo et al. (2020) [109] analyzed feeding and drinking behavior patterns, achieving up to 96.5% accuracy in estrus prediction. Wongvivatvaitaya et al. (2023) [110] further enhanced prediction performance by combining neck temperature measurements with motion data using supervised learning models.

Recent advancements have integrated thermal imaging with deep learning for improved automation. For example, systems combining infrared imaging with object detection models enable real-time monitoring and detection of estrus-related temperature changes in farm environments. Overall, thermal imaging provides a complementary approach to vision-based behavioral analysis by incorporating physiological indicators, thereby improving the robustness and accuracy of estrus detection systems. However, factors such as environmental temperature variation and sensor cost may influence system performance and adoption. Table 4 summarizes the cattle estrus detection method using computer vision.

### 3.5. Drinking and Feeding Detection

Drinking and feeding behaviors are vital in cattle management, impacting health, welfare, and productivity. Cattle typically drink 3–5 times daily, with each drinking bout lasting 1–4 min, influenced by factors such as body weight, milk production, diet, and environmental conditions [113]. Similarly, cattle spend approximately 4–6 h per day feeding, spread across 9–14 meals, with feeding activity typically peaking around dawn and dusk [114]. Feeding is often a group activity where cattle synchronize their eating times, and critical measures include feeding time, meal frequency, and feeding rate. Traditional manual methods to detect these behaviors include visual observation, stopwatches, and jaw movement observation. Computer vision has emerged as a powerful tool for monitoring drinking and feeding behaviors in cattle, offering an automated and non-invasive approach to behavior tracking. Using cameras installed near water stations or feed bunks, computer vision systems can capture footage of cattle and apply algorithms to analyze the footage for behaviors.

Computer vision-based methods for detecting cattle drinking and feeding behaviors can be broadly categorized into behavior detection through head and body position analysis, image-based feed intake monitoring, and deep learning-based classification techniques. Behavior detection methods use camera systems to analyze the positioning and movement of cattle’s heads and bodies, identifying specific behavioral patterns associated with drinking and feeding. Image-based feed intake monitoring approaches quantify actual feed consumption through changes detected in feed piles before and after feeding events using depth imaging and CNN models. Advanced deep learning classification methods enhance detection accuracy by employing neural network architectures to recognize behavioral and differentiate activities such as feeding from non-feeding behaviors. Most studies targeted dairy cows, with reported accuracies for drinking and feeding behavior detection ranging from 81.73% to 97.35%.

#### 3.5.1. Behavior Detection Using Head and Body Position

Several studies have utilized video-based models to monitor feeding and drinking behaviors by analyzing the position and movement of a cow’s head and body. Ref. [115] developed a system using two cameras to monitor dairy calves’ head positions relative to feeding and drinking basins. The program recognized feeding or drinking based on head movements into specific zones, with motion tracking and classification performed in MATLAB. Similarly, Islam et al. (2023) [116] employed DeepLabCut, a pose estimation tool with a ResNet50 backbone, to track body parts such as the head, neck, and ears. These tracked points were analyzed using an LSTM network to classify drinking behaviors based on subtle movements.

Ali Salah (2024) [117] combined YOLO object detection and EfficientNet for a two-step system to identify drinking behavior in cows. YOLO detected the presence of cows at the water station, and EfficientNet classified their posture and position to determine if they were drinking. Meanwhile, Guo et al. (2023) [118] used YOLOv5s-CA with DeepSORT-ViT to track individual cow faces, identifying feeding behavior by observing the position of the face relative to the feed trough. This robust model handled challenges like overlapping animals and occlusions effectively.

#### 3.5.2. Feed Intake Monitoring Through Image Analysis

Monitoring feed intake using video footage is another common approach. Saar et al. (2022) [119] employed overhead cameras to capture images of feed piles before and after meals, using CNNs with transfer learning to analyze the amount of food consumed. Similarly, Wang et al. (2023) [120] applied a Siamese network model with depth imaging to compare food piles before and after feeding events, enabling precise estimation of feed consumption. The system captured subtle changes in the feed pile by analyzing depth patterns rather than simple subtractions.

Earlier systems, such as Porto et al. (2015) [121], used simpler methods by analyzing the position of cows relative to feed barriers in overhead images. The system identified feeding behavior based on whether cows stood with their heads through the barrier. Bezen et al. (2020) [122] enhanced this by incorporating RGB-D cameras to observe changes in depth and shape in the feed pile, employing CNNs to differentiate between feeding and drinking.

#### 3.5.3. Deep Learning for Feeding Behavior Detection

Advanced deep learning models have significantly improved the precision of feeding behavior detection. Bresolin et al. (2023) [123] employed YOLOv3 to monitor heifers at feeding rails, achieving 96% accuracy. The system extracted metrics such as the number of feeding visits, mean visit duration, and total feeding time by detecting head movements in the feed rail zone. Yu et al. (2022) [124] introduced DRN-YOLO, an enhanced YOLO-based model with a DenseResNet backbone and Spatial Pyramid Pooling, to distinguish between feeding and non-feeding activities based on head positions. Achour et al. (2020) [125] took a multi-CNN approach to identify feeding behaviors by classifying head positions as feeding or standing and assessing food availability in the feeder zone. The system only labeled cows as feeding when food was visible, combining CNNs for presence detection, head position classification, and feed analysis. Table 5 summarizes the cattle drinking and feeding detection method using computer vision.

### 3.6. Breathing Detection

Cattle breathing, also known as respiration rate, is a key indicator of an animal’s health status. The standard respiration rate for cattle typically varies depending on age, activity level, and environmental conditions. Respiration in cattle involves flank movements as they inhale and exhale. Observing these flank movements is the most common technique used to measure respiration rate [127]. While this manual method can be effective, it is often time-consuming and labor-intensive. As a result, researchers are now developing automated systems that use cameras and computer vision methods to monitor cattle’s breathing more efficiently.

Automated methods for respiration rate detection in cattle primarily include optical flow-based models, thermal imaging models, object detection techniques, and advanced transformer-based approaches. Optical flow methods track subtle periodic flank movements to measure respiration cycles using algorithms like Horn-Schunck and Lucas-Kanade (LK). Thermal imaging techniques monitor temperature fluctuations around the nostril area, effectively capturing breathing-induced changes in airflow temperature. Object detection models focus on identifying regions that exhibit clear respiratory motion, while transformer-based models leverage self-attention mechanisms to analyze spatiotemporal respiratory patterns in video data dynamically. The majority of breathing detection studies have been conducted on dairy cows, with reported accuracy ranging from 87% to 96.8%.

#### 3.6.1. Optical Flow-Based Models

Optical flow-based models have been widely used for cattle respiration detection by capturing subtle, periodic body movements associated with breathing. These approaches analyze motion patterns in video frames, focusing on the flank or abdominal region where respiratory expansion and contraction are most visible. Wang et al. (2023) [128] introduced a method based on the Horn–Schunck optical flow algorithm to track brightness changes across frames and quantify respiratory motion. By isolating the central body region and applying Fast Fourier Transform (FFT), the system identified dominant respiratory frequencies and accurately estimated respiration rates [129].

Similarly, Wu et al. (2020) [130] used the Lucas–Kanade (LK) optical flow algorithm to analyze motion magnitude and direction after amplifying subtle respiratory signals with phase-based video magnification (PBVM). This approach captured periodic directional changes in motion, enabling the identification of complete respiratory cycles [130].

More recent work has integrated optical flow with object detection and tracking. Shu et al. (2024) [131] combined YOLO-based detection with Lucas–Kanade optical flow to isolate the flank region and extract motion signals, which were further analyzed with FFT to estimate respiration frequency [131]. Overall, optical flow-based methods provide a non-contact, relatively simple solution for respiration monitoring; however, their accuracy can be affected by noise, animal movement, and environmental disturbances.

#### 3.6.2. Thermal Imaging Model

Thermal imaging has emerged as a robust technique for monitoring cattle respiration by capturing temperature fluctuations associated with inhalation and exhalation. During breathing, cooler inhaled air and warmer exhaled air create measurable temperature variations around the nostrils that can be tracked over time. Kim and Hidaka (2021) [132] developed a thermal imaging system using Mask R-CNN to detect and isolate the nostril region, enabling accurate tracking of temperature changes within a defined region of interest [132]. Zhao et al. (2023) [133] further demonstrated that thermal cameras can effectively capture respiratory cycles by monitoring periodic temperature increases and decreases near the nostrils [133].

Recent advancements have improved the robustness of thermal-based approaches through deep learning integration. Chen et al. (2025) [134] employed YOLOv8-Pose for precise nostril localization and combined it with machine learning techniques to generate stable respiratory signals, even under head movement [134]. Similarly, Kim et al. (2026) [135] utilized Mask R-CNN to track nostril temperature patterns and detect specific respiratory events, such as post-regurgitation deep inhalation, providing a physiologically meaningful assessment of respiration [135]. Overall, thermal imaging offers a highly reliable and non-invasive approach for respiration monitoring, although factors such as ambient temperature variation and sensor cost may influence practical deployment. 

#### 3.6.3. Object Detection Models

Object detection-based approaches have become increasingly common for respiration monitoring by identifying key body regions where breathing movements are most prominent. These models typically focus on regions such as the flank, abdomen, or head and track subtle motion patterns over time. Zeng et al. (2023) [136] modified YOLOv5 to detect the head and trunk of calves and incorporated frame-difference features to capture subtle breathing-related motion changes [136].

Recent studies have extended this approach by integrating spatiotemporal modeling. Wang et al. (2024) [137] applied a transformer-based model, VideoMAE, to analyze respiration patterns from RGB video data. By dividing frames into patches and applying self-attention mechanisms, the model effectively captured both spatial and temporal dependencies associated with breathing [137]. Additionally, deep learning-based segmentation and motion analysis methods have improved detection performance. Bhattacharya et al. (2026) [138] used the Segment Anything Model (SAM) to isolate cattle body regions and track shape changes over time, converting these variations into respiratory signals for accurate rate estimation [138]. Similarly, Curti et al. (2025) [139] applied spatiotemporal deep learning models to learn respiration patterns directly from video sequences, enabling automated and scalable respiration monitoring [139]. Overall, object detection and deep learning-based approaches provide a flexible and scalable framework for respiration monitoring, particularly in real-world farm environments with multiple animals and dynamic conditions. Table 6 summarizes the cattle breathing detection method using computer vision.

### 3.7. Behavior Recognition

Accurately recognizing cattle behaviors such as feeding, lying, mounting, and fighting is essential for monitoring animal welfare, health, and productivity in livestock management. Traditional observation methods are limited by their labor-intensive and subjective nature. Therefore, advanced computer vision and sensor-based technologies have emerged to provide efficient, automated, and objective assessments of cattle behaviors, significantly enhancing management practices.

Behavior recognition studies have primarily focused on dairy cows, with reported accuracy rates ranging from 85% to 99.18%. Automated behavior recognition methods typically fall into several categories: YOLO-based object detection models, temporal behavior recognition using 3D convolutional networks, targeted object detection techniques for behavioral analysis, and advanced ResNet-based frameworks. YOLO-based models efficiently track and classify behaviors using enhanced network architectures, attention mechanisms, and robust tracking algorithms. Temporal recognition models utilize specialized 3D convolutional neural networks to effectively capture dynamic behaviors over time. Object detection models specifically analyze essential anatomical landmarks to identify behaviors through posture and movement patterns. ResNet-based models leverage deep residual learning architectures and optimized training methods to robustly classify distinct behaviors from high-resolution images.

#### 3.7.1. YOLO-Based Models

YOLO-based models have become a dominant approach for automated cattle behavior recognition because of their real-time detection and robustness in complex farm environments. Li et al. (2024) [143] developed an enhanced YOLOv8 framework that integrates DSConv modules and BiFormer attention mechanisms to accurately classify behaviors such as standing, lying, eating, and mounting under varying lighting and density conditions. To ensure consistent identification of individual animals, the system incorporated a ResNet18-based re-identification network combined with DeepSORT tracking, effectively reducing identity mismatches in crowded scenes.

Similarly, Tong et al. (2024) [144] improved YOLOv8 by integrating DyConv and C2f-iRMB structures, enabling detection of both common and less frequent behaviors, such as crawling and fighting, while addressing challenges like occlusion and illumination variability. Recent advancements have further enhanced YOLO-based frameworks by combining detection with behavior tracking and identity recognition. Mon et al. (2024) [145] integrated YOLOv8 detection with tracking and feature extraction models to monitor individual cattle behavior patterns over time, improving robustness in real-world conditions.

Additionally, Yu et al. (2024) [146] proposed an improved YOLO-based architecture (Res-DenseYOLO) with attention mechanisms and dense connections to enhance recognition accuracy for behaviors such as feeding, drinking, and resting. Overall, YOLO-based models provide an efficient and scalable framework for real-time behavior recognition, particularly in dynamic farm environments with multiple animals.

#### 3.7.2. Temporal Behavior Recognition

Temporal behavior recognition methods capture dynamic patterns of cattle movement over time, enabling more accurate classification of behaviors that single-frame analysis cannot fully describe. Ma et al. (2022) [147] employed a Rank Expansion Network (RexNet 3D) to classify behaviors such as lying, standing, and walking by extracting spatiotemporal features from video data. By extending traditional ResNet architectures with modules such as ConvBNSwish and SENet recalibration, the model improved the representation of both spatial and temporal features, outperforming conventional architectures such as ResNet101 and MobileNetV2.

Recent studies have further incorporated sequence modeling techniques to analyze long-term behavioral patterns. Mg et al. (2025) [148] used trajectory-based movement data combined with Long Short-Term Memory (LSTM) networks to identify behavioral changes associated with pre-calving events, demonstrating the importance of temporal analysis in detecting subtle behavioral transitions. These approaches highlight the significance of temporal modeling in understanding complex behavioral patterns, particularly for the early detection of physiological and health-related changes.

#### 3.7.3. Object Detection for Behavioral Analysis

Object detection-based approaches are widely used to identify key body parts and positions essential for analyzing cattle behavior. Jiang et al. (2019) [149] proposed the FLYOLOv3 model to detect critical anatomical regions such as the head, back, and legs, enabling the extraction of posture-related features for behavior analysis. The model incorporated a FilterLayer to reduce noise and improve detection accuracy in visually cluttered farm environments.

More recent systems have combined detection with multimodal data sources. Peng et al. (2024) [150] integrated inertial measurement unit (IMU) data with machine learning models to classify behaviors such as feeding, lying, and scratching by transforming motion signals into image-like representations for pattern recognition. Additionally, Giannone et al. (2025) [151] used YOLO-based detection combined with region-of-interest analysis to monitor feeding behavior by tracking the time cows spend in designated feeding zones, providing insights into animal health and welfare. These methods demonstrate that accurate detection and localization of cattle body regions form the foundation for reliable behavioral analysis.

#### 3.7.4. ResNet-Based Models

ResNet-based models are widely used for cattle behavior classification because of their strong feature extraction and robustness in complex visual environments. Cheng et al. (2023) [152] applied a ResNet-based framework to classify behaviors such as standing, walking, lying, and looking back, using high-resolution images and data augmentation to improve performance under challenging farm conditions.

The study compared architectures, including ResNet18 and ResNet50, and found that deeper networks, such as ResNet50, achieved higher accuracy in distinguishing subtle behavioral differences. Training strategies, such as stochastic gradient descent (SGD) with optimized learning rates, further enhanced model performance.

More recent work has integrated ResNet backbones into hybrid systems. Negreiro et al. (2026) [153] used a Siamese neural network with a ResNet-based backbone to track individual cattle and analyze behavior patterns over time, demonstrating the effectiveness of combining feature extraction with identity recognition. Overall, ResNet-based approaches remain a strong baseline for behavior classification and are often integrated with detection and tracking models to build comprehensive behavior monitoring systems. Table 7 summarizes the cattle behavior recognition method using computer vision.

### 3.8. Vision Sensors and Deployment Configurations

**Figure 2:**
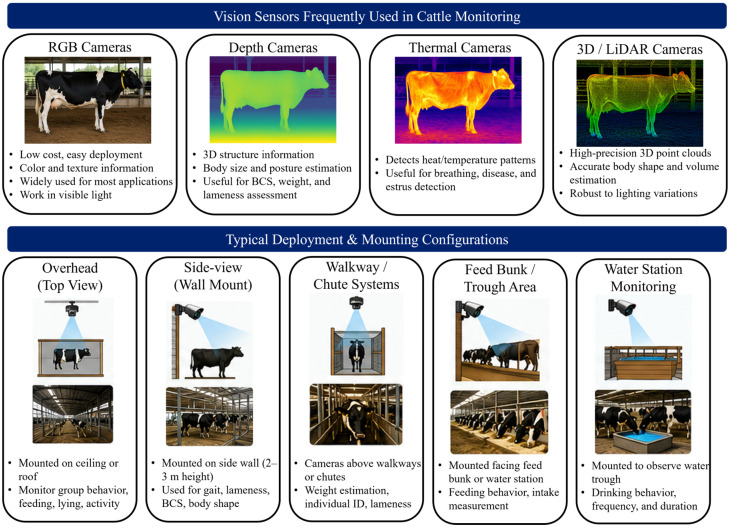
Frequently used vision sensors and typical deployment configurations in cattle health and welfare monitoring.

The effectiveness of computer vision systems for cattle health and welfare monitoring depends not only on the algorithms employed but also on the selection of appropriate vision sensors and deployment configurations. Figure 2 summarizes the most commonly used vision sensors and their typical installation locations in cattle production environments.

RGB cameras are the most widely used imaging devices because of their low cost, ease of deployment, and ability to capture color and texture information. They are commonly used for behavior monitoring, estrus detection, feeding and drinking behavior analysis, and general animal observation. Depth cameras provide three-dimensional structural information, enabling body size measurement, posture analysis, BCS, weight estimation, and lameness assessment. Thermal cameras capture surface temperature distributions and are frequently used for respiration monitoring, disease detection, and estrus identification. LiDAR and other 3D imaging systems generate detailed point clouds that support accurate characterization of body shape and volume-based weight estimation.

Camera placement varies depending on the monitoring objective. Overhead camera systems are commonly installed on ceilings or roofs to monitor group behavior, activity, feeding, and lying patterns. Side-view cameras are frequently used for gait analysis, lameness detection, body condition scoring, and body shape assessment. Cameras positioned above walkways or chutes facilitate individual animal identification, weight estimation, and locomotion analysis. Feed bunk and water station monitoring systems are designed to quantify feeding and drinking behavior, intake patterns, and resource utilization. The selection of sensor type and deployment configuration should therefore be aligned with the specific health or welfare trait being monitored.

Beyond sensor selection, successful deployment of computer vision systems requires consideration of practical factors such as camera placement, lighting conditions, animal identification and tracking, and occlusion caused by animals, feeding equipment, or facility structures. Long-term operation may also be affected by dust, moisture, environmental variability, and maintenance requirements. Therefore, sensor selection and deployment configuration should be aligned not only with the target health or welfare trait but also with the operational conditions of the production environment to ensure reliable and actionable monitoring outcomes.

## 4. Discussion

**Figure 3:**
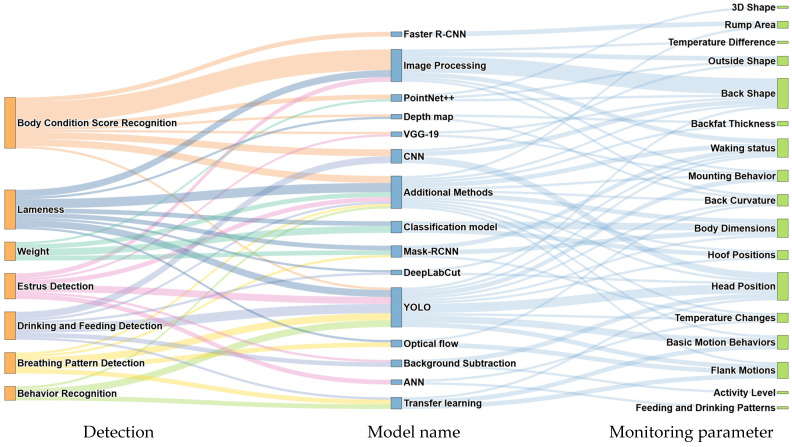
Relationship of detection techniques, tools, and monitoring parameters for cattle health and welfare monitoring.

The adoption of computer vision technologies in cattle farming represents an idea of how the health and welfare of livestock are monitored and managed. The findings from this review highlight the transformative potential of computer vision tools in addressing key challenges in cattle farming, such as labor inefficiencies, scalability, and animal health monitoring, while promoting better decision-making and sustainability. Figure 3 summarizes the current computer vision tools and monitoring parameters for cattle health and welfare monitoring.

Based on the findings and insights from this review, the discussion highlights some key areas where computer vision technologies have significantly impacted cattle farming. These areas address challenges in traditional practices, enhance productivity, promote animal welfare, and contribute to sustainable agricultural systems. Each area offers a focused perspective on how these technologies shape modern livestock management.

The review paper is trying to address six guiding questions that help frame both current applications and future directions. The first four questions focus on what has already been demonstrated in the field, addressing the challenges that computer vision has tackled, the types of imaging systems used for data collection, the algorithms and models applied, and the specific techniques that have achieved the best performance. These sections summarize the state of the art and illustrate how existing tools contribute to enhanced decision-making and management in cattle farming.

The final two questions highlight the ongoing challenges that hinder broader adoption, such as environmental variability, data limitations, and infrastructure constraints, while also identifying underexplored areas of cattle health and welfare that could benefit from current computer vision capabilities. These insights aim to inform future research and innovation, promoting the adaptation of proven technologies to new and impactful applications in precision livestock farming.

### 4.1. RQ1: What Specific Issues in Cattle Farming Have Been Addressed Using Computer Vision Technologies?

Computer vision has been applied to a wide range of challenges in cattle farming, particularly in enhancing health and welfare. It has been utilized to estimate feed intake, monitor body weight and gain, assess body condition score, evaluate health status, and track reproductive events. One of the key advantages of computer vision is its capacity to provide early warnings for health issues by automatically detecting signs of illness or discomfort. Applications such as lameness detection through gait and posture analysis enable timely intervention and disease management.

Computer vision technologies also play a significant role in behavior recognition related to both welfare and reproduction. Automated video analysis can monitor feeding and drinking behaviors, social interactions, and reproductive actions such as mounting, which is a key indicator of estrus. Tracking these behaviors allows for a better understanding of activity patterns, estrus timing, and overall well-being, while also assisting in detecting signs of distress or illness through unusual behaviors. Additionally, object detection algorithms are employed for animal counting and tracking, enabling the distinction of cattle from the background and counting individuals in groups. This supports herd management by monitoring distribution in grazing areas, checking for missing animals, and analyzing facility usage. Computer vision has also facilitated non- invasive individual identification through unique biometric traits such as coat patterns, facial features, and muzzle prints. High- accuracy models allow for consistent tracking of individual animals, linking visual identity to performance and health records without the need for physical tags.

Another notable application of computer vision is in evaluating body condition score and estimating weight. These tasks are accomplished by analyzing images of the cow’s body shape and fat distribution or using stereo and depth cameras to assess body dimensions. This enables accurate, non-contact monitoring of nutritional and growth status, supporting improved feeding strategies and market timing decisions. Overall, computer vision technologies are transforming cattle farming by providing continuous, objective, and automated monitoring solutions that enhance both animal management and welfare.

### 4.2. RQ2: What Types of Data Acquisition Systems Have Been Used in Computer Vision Applications for Cattle Farming?

Successful computer vision applications in cattle farming depend on robust data collection systems that can capture images or videos of animals in their surroundings. Various imaging setups have been implemented to address this. Fixed camera arrangements are often placed strategically on farms for ongoing monitoring. This includes overhead cameras mounted on barn ceilings or at entry and exit points and side-view cameras positioned in feeding or milking areas. These fixed installations provide the animals’ top, side, or frontal perspectives and are typically linked to network video recorders that continuously capture footage. They can monitor behaviors such as feeding and resting, track movement through alleys, and detect events when cows pass specific locations. Additionally, cameras mounted on autonomous robots or rail systems can navigate through barns, taking close-up images of individual cows.

In addition to conventional RGB cameras, advanced imaging technologies have been utilized to obtain more detailed and varied data. Depth cameras, using stereo vision or structured light, capture 3D shape information, which helps estimate body dimensions or weight. Meanwhile, thermal infrared cameras identify temperature differences, aiding in detecting illness signs, such as breathing or heat stress. Computer vision systems enhance accuracy by combining different imaging data such as color, depth, and thermal. For instance, integrating RGB visuals with thermal data can assist in pinpointing animals with higher body temperatures. In summary, data acquisition for cattle computer vision ranges from fixed surveillance cameras in controlled settings to mobile robots, gathering imagery from various perspectives and spectral ranges. This diverse input is crucial for effective computer vision analysis. Choosing the right camera type and ensuring comprehensive herd coverage are vital initial steps for developing successful computer vision solutions in cattle farming.

### 4.3. RQ3: Which Computer Vision Techniques and Algorithms Have Been Implemented in the Context of Cattle Farming?

A wide range of computer vision techniques, from traditional image processing to advanced deep learning algorithms, have been implemented in cattle farming to analyze animal’s health and welfare effectively. Over time, the field has increasingly shifted toward deep neural networks due to their higher accuracy and robustness in complex farm environments. Early methods used conventional image processing and machine learning approaches, utilizing hand-crafted features and classical classifiers. Techniques like background subtraction and contour detection were employed to isolate animals, with features such as shape and texture input into models like support vector machines or decision trees. While these methods achieved some success, they often struggled under variable and uncontrolled farm conditions, such as fluctuating lighting, moving backgrounds, and occlusions.

To address these challenges, more recent applications have turned to deep convolutional neural networks, particularly for object detection and segmentation tasks. One-stage detectors like YOLO and Single Shot Detector and two-stage models like Faster R-CNN have been widely adopted to locate cattle or their body parts in images. These models can automatically learn features and draw bounding boxes around animals, even in crowded environments. Instance segmentation models, such as Mask R-CNN, provide even more detailed analysis by generating pixel-level masks, enabling precise shape detection and separation of overlapping animals. Systems often combine speed and accuracy by using fast detectors like YOLO for real-time tracking and Mask R-CNN for detailed segmentation.

Image classification CNNs, such as VGG16, ResNet-50, and DenseNet, were initially developed for general image recognition. DenseNet has been applied to detect skin lesions associated with diseases like lumpy skin, achieving extremely high accuracy. Other studies have trained CNNs to classify body condition scores from side-profile images or to detect lameness by analyzing gait patterns. These deep learning models outperform earlier methods by automatically learning subtle, task-specific features, such as the curvature of a cow’s back or signs of swelling in hooves.

Pose estimation and tracking algorithms represent another critical category of computer vision techniques in cattle farming. These approaches detect key anatomical points on the animal’s body, such as joints and spine landmarks, and track them over time to assess posture and movement. Frameworks like DeepLabCut and OpenPose have been adapted for cattle to study gait and lameness. In some cases, models like Mask R-CNN have been modified to include key point detection for posture analysis. When combined with tracking algorithms such as SORT, these methods enable frame-by-frame analysis of animal movement. Additionally, temporal modeling techniques, such as LSTM networks, have been layered on top of CNN feature extractors to analyze behavior sequences over time. For instance, a YOLO-based model might detect cows in each video frame, while an LSTM analyzes the motion patterns to classify actions like mounting or lying down.

In summary, computer vision in cattle farming has advanced from basic image processing to sophisticated deep learning models. Object detection and segmentation networks manage localization, classification models support identification and health assessment, and pose estimation techniques facilitate behavioral and gait analysis. These approaches are often integrated into comprehensive systems adapted to specific monitoring tasks, enabling more accurate and automated cattle health and welfare management.

### 4.4. RQ4: Which Computer Vision Techniques Have Demonstrated the Highest Performance in Addressing Specific Challenges in Cattle Farming?

Different computer vision approaches tend to excel in specific cattle-related tasks, and researchers have identified the most effective techniques for each application. For animal detection and counting, deep learning-based object detectors have demonstrated excellent performance. One-stage CNN detectors like YOLO and SSD are particularly effective and highly accurate. These models achieved detection precisions above 90% in farm environments, making them ideal for counting cattle in pens or aerial imagery. Two-stage detectors such as Faster R-CNN can deliver slightly higher accuracy. However, the YOLO family is often preferred for on-farm deployment due to its balance of speed and accuracy. Computer vision is emerging as a powerful diagnostic tool in disease detection and health monitoring. High-performing models, such as DenseNet-201 enhanced with attention mechanisms, have attained around 97% accuracy in identifying cattle with skin lesions caused by diseases like lumpy skin.

Lameness and gait abnormality detection is another area where computer vision has made significant strides. The most accurate approaches utilize a combination of object detection, pose estimation, and classification. One notable system used Mask R-CNN to extract body posture and a classifier to analyze gait, achieving 94–100% accuracy in identifying different degrees of lameness. This level of performance highlights the advantage of employing deep pose estimation and temporal models, such as LSTMs, which track motion over time and enhance behavior classification.

Estimating BCS and weight from images is complex, but recent models have considerably reduced errors. Traditional methods often had error margins of 8–10%, but by using depth cameras, multi-view imaging, and deep regression networks, researchers have lowered weight prediction errors to around 3%. For instance, in a 350 kg cow, this equates to a 10 kg error, which is within acceptable limits for practical use. CNN-based BCS classification models also show high agreement with expert scores, frequently surpassing 90% accuracy. The best results come from combining 3D information, like point clouds or multiple 2D perspectives, enabling more precise body shape and fat coverage analysis.

In summary, the most advanced computer vision systems in cattle farming are typically deep learning-based and tailored to specific tasks. Object detectors like YOLO and Faster R-CNN excel in locating and counting cattle, identification models achieve near-perfect accuracy through detailed visual features, pose estimation and tracking models effectively flag gait abnormalities, and specialized CNNs provide quantitative insight into body condition and disease. These technologies enable a new level of precision and automation in cattle health and production monitoring.

### 4.5. RQ5: What Challenges Are Faced When Applying Computer Vision Techniques in Cattle Farming?

Implementing computer vision in real-world cattle farming presents a range of challenges that researchers and producers must navigate. One major issue is environmental variability and image quality. Barns and pastures introduce inconsistent conditions, with lighting varying widely from bright daylight to dim interiors, and backgrounds often covered with animals, equipment, or moving shadows. Additionally, cows frequently move in groups or occlude one another, while camera angles may be suboptimal or obstructed. These factors complicate image interpretation and reduce the reliability of detection, tracking, and identification algorithms.

Another significant hurdle is data scarcity, and the effort required for annotation. Deep learning models typically need large, labeled datasets for effective training, but such resources are scarce in agriculture due to privacy concerns, high data collection costs, and limited public datasets. While transfer learning using models pre-trained on general datasets like ImageNet offers some help, the domain gap between general images and farm-specific visuals limits its efficiency. Moreover, creating high-quality annotations, such as labeling key points or segmenting animals in images, requires considerable labor, often from domain experts. As a result, the lack of annotated data represents a significant bottleneck in developing generalizable and high-performing models.

The deployment also brings hardware and infrastructure challenges. Cameras must be placed strategically, maintained regularly, and be resilient to harsh barn conditions such as dust, humidity, and temperature fluctuations. Covering all critical areas often requires multiple cameras, raising costs and maintenance complexity. Additionally, many farms lack bandwidth and computing resources to support real-time deep-learning inference. High-end GPUs or reliable cloud access are often unavailable, necessitating the optimization of models for edge devices or offline processing. Ensuring reliable power, networking, and hardware stability remains challenging in these environments.

Interdisciplinary knowledge gaps further complicate computer vision implementation. Developing effective systems necessitates close collaboration between computer scientists and domain experts like animal scientists or veterinarians. Tasks such as detecting disease symptoms, assessing body condition scores, or interpreting behavioral cues demand specific domain knowledge that is not typically within the purview of engineers. Conversely, individuals with agricultural expertise may be unfamiliar with the capabilities and limitations of AI technologies, which can lead to miscommunication, misaligned expectations, or poorly designed systems. The quality of data labeling also relies on expert input, such as accurately identifying complex behaviors.

Real-time processing and reliability are critical in practical operations. On farms, computer vision systems often need to operate in real time to provide timely alerts, such as signaling distress or abnormal behavior. However, deep learning models are computationally demanding, making fast inference on low-power hardware challenging. Lightweight models like Tiny-YOLO or MobileNet offer speed but may compromise accuracy, while more complex models provide higher precision but may be too slow for real-time use. Striking the right balance between performance and efficiency remains an ongoing research focus. Simultaneously, systems must be reliable by minimizing false positives and missed detections to gain farmers’ trust and ensure actionable insights.

In summary, the practical application of computer vision in cattle farming is challenged by environmental unpredictability, limited and labor-intensive data availability, hardware constraints, the need for interdisciplinary collaboration, and real-time performance demands. These issues help explain why some promising computer vision solutions remain confined to research settings rather than being widely adopted on commercial farms. Addressing these obstacles through improved datasets, robust model training, hardware adaptation, and closer collaboration across disciplines is key to making computer vision a reliable tool for everyday cattle management.

### 4.6. RQ6: How Could Underexplored Health and Welfare Aspects Benefit from Utilizing Existing Computer Vision Models in Cattle Farming?

Computer vision applications in cattle health and welfare monitoring have observed outstanding progress in recent years, using advances in machine learning and deep learning algorithms. However, several critical challenges remain unaddressed, offering significant future research and development opportunities. One such challenge is the detection of disease-specific indicators. While current systems proficiently identify general health issues like lameness, they often lack the sensitivity to detect subtle signs of specific diseases. For instance, early-stage mastitis, a common and economically significant illness in dairy cattle, establishes itself through subtle changes in udder shape and skin temperature [167,168]. By integrating thermal imaging and high-resolution cameras, computer vision systems could enhance diagnostic accuracy by capturing these nuanced physiological changes. Advanced image processing techniques, such as those provided by OpenCV, could analyze nasal discharges that signal respiratory diseases [169], while segmentation models like Mask R-CNN and transfer learning could identify skin lesions indicative of parasitic infestations [170]. These enhancements would facilitate early intervention, improve animal health outcomes, and reduce economic losses for farmers.

Another promising boundary is the assessment of emotional and cognitive states, which are essential yet often underappreciated aspects of cattle welfare. Cattle, like other sentient beings, exhibit emotional responses that can indicate their well-being [171]. Computer vision systems can detect signs of stress, pain, or fear by analyzing facial expressions—including ear positioning, eye tension, and muscle movements. Integrating vocalization patterns could provide a multidimensional understanding of distress signals, enabling more proactive and compassionate interventions. Deep learning algorithms, such as CNNs and YOLO, which have already demonstrated efficacy in tracking specific body parts like heads and flanks, could be adapted to assess these subtle behavioral cues.

Calving and the postpartum period represent critical times for the cow and the calf, with significant implications for their health and welfare. Current monitoring systems are limited in their ability to automate the detection of early labor signs and postpartum recovery indicators. By extending the capabilities of existing models used in activity level analysis and motion detection, such as those employed in breathing pattern and body condition monitoring, computer vision systems could predict the onset of labor and monitor postpartum behaviors [172]. Techniques like background subtraction and 3D imaging could track maternal bonding and calf mobility, ensuring timely interventions when necessary.

Open grazing systems present additional challenges, as most current computer vision applications are designed for controlled environments like feedlots. Future innovations could focus on monitoring individual grazing behaviors, such as time spent grazing, bite frequency, and vegetation preferences [3]. Techniques like optical flow and quantile regression, previously applied in feeding behavior detection, could be adapted for open field conditions, providing insights into foraging strategies and dietary intake.

Addressing heat stress is another area where computer vision can expand its utility. Traditional systems often rely on environmental measurements, which may not accurately reflect the animal’s thermal discomfort [173]. By detecting behavioral and physical signs, such as shade-seeking behavior, excessive panting, or drooling, computer vision systems could offer more direct assessments of heat stress. Advanced object detection methods like Faster R-CNN could be employed to capture these behaviors, allowing for timely interventions to mitigate stress and maintain productivity.

In mixed-species farming systems, monitoring interspecies interactions is crucial for understanding resource competition, social behaviors, and disease transmission risks [174]. Computer vision could be employed to analyze these dynamics, providing valuable insights into the complexities of multi-species environments. Robust object detection capabilities, including models like YOLO and Faster R-CNN, could track different species simultaneously, aiding in the management of shared resources and minimizing conflicts or disease spread. This could lead to more harmonious cohabitation and efficient use of resources, benefiting the overall farm ecosystem.

Environmental sustainability, increasingly linked with cattle management practices, could also benefit from advancements in computer vision [175]. Monitoring manure distribution and grazing intensity would enhance nutrient cycling and pasture health, promoting more sustainable agricultural practices [176]. These environmental assessments align with global sustainability goals and can help farmers meet regulatory requirements or consumer expectations for environmentally responsible farming. A possible solution for manure detection involves integrating computer vision models tailored for specific tasks. YOLO, a real-time object detection algorithm, could be employed to detect and localize manure piles in diverse environments effectively. For more detailed analysis, segmentation models like DeepLabV3+, UNet, or SAM can segment manure areas precisely, providing spatial distribution data critical for understanding grazing patterns and nutrient cycling.

In terms of environmental impact, visual recognition of eructation frequency could estimate methane emissions, while manure distribution detection would inform pasture health. Integrating pose estimation techniques like DeepLabCut could address challenges in monitoring cattle in dense herds or low visibility conditions. DeepLabCut enables precise, markerless tracking of cattle poses, detecting subtle behavioral or postural changes indicative of health issues or stress. Combined with night vision, it ensures continuous monitoring, reducing missed indicators due to poor lighting. This approach enhances grazing analysis, social behavior tracking, and early disease detection, supporting welfare and sustainability goals.

## 5. Conclusions

Integrating computer vision technologies into cattle health and welfare monitoring has revolutionized traditional livestock management practices, offering more accurate, efficient, and non-invasive solutions. Advanced imaging systems combined with artificial intelligence techniques have enabled automated extraction of body measurements, posture characteristics, locomotion patterns, feeding and drinking activities, respiratory movements, and behavioral indicators. These capabilities facilitate continuous, objective, and real-time monitoring of individual animals, reducing reliance on subjective visual assessments and improving management efficiency. This monitoring method also reduces stress for cattle, promoting better welfare while supporting sustainable farming practices through environmental monitoring tasks like grazing pattern analysis and manure distribution tracking.

Despite these advancements, challenges such as variability in environmental conditions, the need for large, annotated datasets, and the high cost of implementation persist. Addressing these issues requires future research to develop robust, cost-effective, and accessible technologies alongside expanding capabilities for disease-specific monitoring, emotional state assessment, and adaptation to open grazing systems.

Overall, the reviewed studies demonstrated promising performance for cattle health and welfare monitoring; however, several methodological limitations were identified. Many studies relied on relatively small numbers of animals despite reporting large image datasets, which may increase the risk of overestimating model performance. External validation across farms, breeds, seasons, and camera systems was rarely conducted, limiting the generalizability of reported results. In addition, reporting of annotation reliability, strategies for addressing class imbalance, and dataset availability was often inconsistent. Another important challenge for video-based monitoring systems is maintaining robust performance under practical farm conditions. Variations in camera frame rate, animal position, viewing angle, and occlusion can influence the accuracy. Therefore, although high accuracies were frequently reported, further validation under diverse commercial production conditions is needed before large-scale implementation can be fully justified.

Computer vision technologies have immense potential to transform cattle farming by fostering a more sustainable, welfare-oriented, and efficient approach to livestock management. With continued innovation and collaboration among researchers, technologists, and industry stakeholders, these tools can significantly contribute to meeting the growing demands of the global cattle industry while promoting animal health and welfare.

## Figures and Tables

**Table 1:** Summarizes the cattle body condition score method using computer vision.

Cattle Type	AI Subset	Measurement	Input	Model Name	Number of Animals	Model Performance	Reference
Dairy Cow	Deep Learning	Outside shape	Image	Depth map	115	Accuracy = 89.00%	[32]
Dairy Cow	Deep Learning	Different locations with different cameras	Image	CNN	50	Accuracy = 97.50%	[33]
Dairy Cow	Deep Learning	Back posture shape	Image	CNN	1700	Accuracy = 94%	[21]
Dairy Cow	Deep Learning	Back posture shape	Image	CNN, Transfer Learning	1661	Accuracy = 97%	[34]
Dairy Cow	Deep Learning	Back posture shape	Image	3D surface map	97	R^2^ = 0.8336	[35]
Dairy Cow	Machine learning	Outside shape	Image	MATLAB (2017b) image processing	44	Sensitivity = 72%	[20]
Dairy Cow	Deep learning	Backfat thickness	Video	DenseNet	686	Accuracy = 98%	[24]
Dairy Cow	Deep learning	Rump shape	Video	Faster R-CNN	1500	Accuracy = 71%	[36]
Dairy Cow	Deep learning	Back	Image	VGG-16	1500	mAP = 98.46%	[25]
Dairy Cow	Machine learning	Back	Image	YOLOv2	2500	Accuracy = 94.50%	[37]
Dairy Cow	Deep Learning	Back posture shape	Image	MATLAB image processing	55	R^2^ = 0.63	[38]
Dairy Cow	Deep learning	Back	Image	VGG-19	10	accuracy = 94.69%	[39]
Dairy Cow	Deep learning	Back limbs and tails status	Image	GMM Model	1000	Accuracy = 94.2%	[29]
Dairy Cow	Deep Learning	Back posture shape	Image	Regression	52	MAE = 0.13	[40]
Dairy Cow	Deep Learning	Back posture shape	Image	Image processing	94	Accuracy = 95.48%	[41]
Dairy Cow	Deep learning	Back	Image	CNN	209	Accuracy = 97.84%	[26]
Dairy Cow	Deep learning	Back posture shape	Image	Regression	526	Accuracy = 78%	[31]
Dairy Cow	Deep Learning	Dorsal, Lateral, Udder, Chest, Back	Image	SqueezeNet	66	Accuracy = 94.42%	[42]
Beef cow	Machine learning	Rump shape	Image	AdaBoost	39	Accuracy = 90%	[43]
Dairy Cow	Image processing	Back posture shape	Image	CNN	462	89	[44]
Dairy Cow	Deep learning	3d view	Image	Shape analysis	298	94.42	[45]
Dairy Cow	Deep learning	Outside shape	Image	PointNet++	512	96.00	[23]
Dairy cows + Beef cattle	Deep Learning	Back posture shape	Image	YOLACT++	120	Accuracy = 90%	[46]
Dairy Cow	Deep learning	Left and right	Image	EfficientNet, point cloud	77	Accuracy = 91.21%	[30]
Dairy Cow	Deep Learning	Back posture shape	Image	Regression	3817	F1-score = 90.4%	[47]
Dairy Cow	Deep learning	Rump shape	Video	Faster R-CNN	2990	Accuracy = 70%	[48]
Dairy cattle	Deep Learning	Tail region	Image	EfficientNet-B0, YOLO	Not specified	Accuracy = 93.77%	[27]
Dairy cattle	Deep Learning	Back region	Image	YOLOv7 + EfficientID	182	Accuracy = 88%	[49]
Dairy cattle	Deep Learning	Hindquarters/tailhead region	Video	Improved YOLOv5	213	Accuracy = 87.1%	[50]
Dairy cattle	Deep Learning	Tailhead and back region	Image	YOLO	Not specified	Accuracy = 86.9%	[28]
Dairy cattle	Machine Learning	Back and hindquarters via overhead view	Video	CattleEye	103	Accuracy = 98.9%	[51]
Dairy cattle	Deep Learning	Tail region	Image	EfficientNet-B0, YOLO	Not specified	Accuracy = 93.77%	[27]
Dairy cattle	Deep Learning	Back region	Image	YOLOv7 (detection) + EfficientID	182	Accuracy = 88%	[49]
Dairy cattle	Deep Learning	Back, front, top views; mainly tailhead and pelvic region	Image	YOLOv8	56	mAP@0.5 = 43.9%; mAP@0.5:0.95 = 41.0%	[50]
Dairy cattle	Deep Learning	Tailhead and back region	Image	YOLO (YOLOv8-based) with C2f-Star-EMA module + SSLDH lightweight	Not specified	Precision = 86.9%, Recall = 84.1%	[28]

**Table 2:** Summary of cattle lameness detection methods using computer vision.

Cattle Type	AI Subset	Measurement	Input	Model Name	Number of Animals	Model Performance	Reference
Dairy Cows	Machine Learning	Back posture	Video	Depth map	511	Accuracy = 90.9%	[73]
Dairy Cows	Deep Learning	Six back curvature features	Video	Depth map	633	Accuracy = 79.8%	[74]
Dairy Cows	Machine Learning	Gait asymmetry	Video	Hilbert Transform, SVM	22	Accuracy = 95.7%	[75]
Dairy Cows	Machine Learning	Gait features (speed, asymmetry)	Video	DT	98	Accuracy = 90.18%	[76]
Dairy Cows	Deep Learning	Supporting phase (hoof ground time)	Video	RFB_Net_SSD	100	Accuracy = 96%	[64]
Dairy Cow	Deep Learning	Lameness detection	Video	Optical flow	90	Accuracy = 98.24%	[60]
Dairy Cows	Deep Learning	Relative step size	Video	YOLOv3, LSTM classification	50	Accuracy = 98.57%	[61]
Dairy Cows	Deep Learning	Lameness detection	Video	YOLOv4, DenseNet	456	Accuracy = 98.5%	[77]
Dairy Cows	Deep Learning	Back curvature	Video	FLYOLOv3	90	Accuracy = 96.61%	[78]
Dairy Cows	Deep Learning	Back curvature	Video	GhostNet_YOLOv4	98	Accuracy = 91.67%	[65]
Dairy Cows	Machine Learning	Lameness detection	Image	CART	33	Accuracy = 93.9%	[62]
Dairy Cows	Deep Learning	Head-hoof and back-hoof linkage	Video	DeepLabCut	52	Accuracy = 89.2%	[68]
Dairy Cows	Deep Learning	Posture and gait	Video	Mask-RCNN, SORT tracking, CatBoost classification	250	Accuracy = 94%	[66]
Dairy Cow	Deep Learning	Lameness recognition	Image	Temporal aggregation network	222	Accuracy = 98.89%	[59]
Dairy Cows	Deep Learning	Temporal gait and spatial pose	Video	SEN using HEI and GEI	330	Accuracy = 96.22%	[79]
Dairy Cows	Deep Learning	17 key points on body	Video	YOLO-based detection, post-processing	34	Accuracy = 89%	[80]
Dairy Cows	Deep Learning	Lameness detection	Video	SOLOv2 Network	246	Accuracy = 98.65%	[71]
Dairy Cows	Deep Learning	Lameness detection	Video	Mask-RCNN and YOLOv8, AdaBoost classification	Not specified	Accuracy = 77.9%	[67]
Dairy Cow	Deep Learning	Lameness detection	Video	T-LEAP pose estimation	98	Accuracy = 80%	[58]
Dairy Cows	Machine Learning	Lameness detection	Video	Depth map	64	Accuracy = 82.3%	[56]
Dairy cattle	Deep Learning	Hoof disease	Images	YOLOv8 + CNN (MobileNetv3/EfficientNet for classification)	160	Accuracy = 91%	[63]
Dairy cattle	Deep Learning	Back shape: cranial, middle, caudal spine regions	Video	DeepLabCut+ (DNN)	260	Accuracy = 97%	[70]
Dairy cattle	Deep Learning	Head, neck/back, mid-back, tail region—keypoints	Video	DeepLabCut+ (CNN + temporal modeling)	45	Accuracy = 90.21%	[69]
Dairy cattle	Machine Learning	Whole-body movement: head, neck, withers, back, hip ridge, tailhead, hooks, pins	Video	keypoint detection	256	Accuracy = 76.0–92.1%	[72]

**Table 3:** Summary of cattle weight estimation method using computer vision.

Cattle Type	AI Subset	Measurement	Input	Model Name	Number of Animals	Model Performance	Reference
Heifers	Machine Learning	Weight, Hip & Withers Height	Depth images	Quantile regression-based ellipse fitting, MLR	107	Accuracy = 94.6%	[83]
Dairy Cows	Machine Learning	Weight Estimation	Images	DT, RF, GB, KNN	104	MAE = 5.45 kg	[89]
Dairy Cows	Deep Learning	Body Dimensions	3D Images	Mask R-CNN, MLP	-	Accuracy = 92%	[84]
Beef Cattle	Deep Learning	Body Weight Estimation	3D Point Cloud	PointNet++ (segmentation) + Johnson’s weight formula	100	Accuracy = 95.1%	[87]
Dairy Cows	Machine Learning	Body Weight, Feed Intake	3D Images	Image processing	9142	Accuracy = 88%	[85]
Dairy Cows	Machine Learning	Body Weight	3D Contour	CatBoost, AdaBoost, Random Forest	914	R^2^ = 0.94	[88]
Dairy Cows	Deep Learning	Body Weight	Depth Images	Mask R-CNN	12	Accuracy = 98%	[86]
Not Specified	Deep Learning	Weight Estimation	Images	ResNet-101-D + SE mechanism, BP Neural Network	55	R^2^ = 0.98	[82]
Beef cattle	Machine Learning + Deep Learning	Body weight	Images	YOLOv8n (+ 3D point cloud	199	R^2^ = 0.85	[90]
Dairy calves	Deep Learning + Machine Learning (hybrid)	Whole body	Depth images	YOLOv8 + body metric extraction (morphometrics)	68	R^2^ = 0.99	[91]

**Table 4:** Summary of cattle estrus detection method using computer vision.

Cattle Type	AI Subset	Measurement	Input	Model Name	Number of Animals	Model Performance	Reference
Dairy Cows	Deep Learning	Mounting behavior	Video	Optical Flow, SVM classifier	10	Accuracy = 90.9%	[96]
Heifers	Machine Learning	Feeding and drinking behavior patterns	Video	ANN	57	Accuracy = 96.5%	[109]
Dairy Cows	Deep Learning	Whole body	Image	Faster R-CNN	17	Accuracy = 90%	[98]
Dairy Cows	Deep Learning	Mounting behavior	Video	SIFT algorithm for image recognition	23	-	[110]
Dairy Cows	Machine Learning	Activity level	Video	Background Image Subtraction	6	F1-score = 0.64, Sensitivity = 90.0%	[97]
Dairy Cows	Deep Learning	Mating posture	Video	Modified YOLOv3 with an additional layer and Mish activation	8	Accuracy = 98%	[102]
Dairy Cows	Deep Learning	Mounting behavior	Video	YOLOv5	200	Accuracy = 94.3%	[99]
Dairy Cows	Machine Learning	Skin temperature and tail movement	Video	Infrared thermography and vulva exposure tracking	47	Accuracy = 76%	[107]
Dairy Cows	Deep Learning	Body part	Image	CowXNet	3	Accuracy = 83%	[101]
Dairy Cows	Thermal Imaging	Temperature of different body parts	Image	LOGISTIC and SVM	10	Accuracy = 82.37%	[108]
Dairy Cows	Machine Learning	Neck temperature and motion data	Temperature feature	ANN, DT, RF	27	Accuracy = 89%	[111]
Dairy Cows	Deep Learning	Mounting behavior	Image	VGG-19 for mounting detection, YOLOv5 for cow identification	300	Accuracy = 94%	[100]
Dairy Cows	Deep Learning	Mounting behavior	Video	YOLOv8n model with NWD loss	1716	Accuracy = 93.9%	[112]
Dairy cattle	Deep Learning	Whole-body activity: walking, flirting, climbing, mating	Image	CNN + Artificial Immune System (AIS) + YOLOv5	10	Accuracy = 98.36%	[103]
Dairy cattle	Deep Learning	Whole-body activity: riding, chin-resting, standing, lying, eating, drinking	Video	YOLOv8	3	F1-score = 0.481	[105]
Dairy cattle	Machine Learning + Deep Learning	Whole-body movement patterns: circling, activity level, direction changes	Video	YOLOv5s (detection) + DeepSORT (tracking)	38 cows	Accuracy = 89.47%	[106]
Dairy cattle	Deep Learning	Whole-body locomotion: standing vs. lying down activity	Video	EfficientPose + YOLOv5	3	mAP@0.5 = 0.80, mAP@0.5:0.95 = 0.80	[104]

**Table 5:** Summary of cattle drinking and feeding detection methods using computer vision.

Cattle Type	AI Subset	Measurement	Input	Model Name	Number of Animals	Model Performance	Reference
Dairy Cows	Machine Learning	Feeding Behavior	Images	Image processing using Viola-Jones	15	Accuracy = 87.00%	[121]
Dairy Cows	Deep Learning	Individual Feed Intake	RGBD Images	CNN	70	Accuracy = 93.65%	[122]
Calf	Deep Learning	Feeding and Drinking Behavior	Video	Otsu’s method of segmenting the image	1	Accuracy = 81.73%	[115]
Dairy Cows	Machine Learning	Feeding Behavior	Images	CNN	17	Accuracy = 92%	[125]
Dairy Cows	Deep Learning	Feed Intake Measurement	RGBD Images	Transfer Learning and CNN	60	MAE = 0.12–0.23 kg	[119]
Dairy Cows	Deep Learning	Feeding Behavior	Video	DRN-YOLO	17	Accuracy = 96.91%	[124]
Beef Cattle	Deep Learning	Drinking Behavior	Video	DeepLabCut for pose estimation; LSTM for classification	4	Accuracy = 97.35%	[116]
Heifers	Deep Learning	Feeding Behavior	Video	YOLOv3	8	Accuracy = 96%	[123]
Dairy Cows	Deep Learning	Feeding Behavior	Video	YOLOv5s-CA for detection, DeepSORT + Vision Transformer	Not specified	Accuracy = 88.5%	[118]
Dairy Cows	Deep Learning	Feed Intake Measurement	Depth Images	Feature difference in depth images	Not specified	Accuracy = 90%	[120]
Dairy Cows	Deep Learning	Drinking Behavior	Video	YOLO for detection, EfficientNetV2-S for classification	12	Accuracy = 88.60%	[117]
Dairy cattle	Deep Learning + Machine Learning	Feeding duration/time at feeder	Images	YOLOv4-tiny+ MobileNetV2	Not specified	F1-score = 0.98	[126]

**Table 6:** Summary of cattle breathing detection method using computer vision.

Cattle Type	AI Subset	Measurement	Input	Model Name	Number of Animals	Model Performance	Reference
Dairy Cows	Machine Learning	Respiration Rate	Non-radiometric IR Videos	KLT algorithm	10	Accuracy = 87%	[140]
Dairy Cows	Deep Learning	Respiratory Rate	Video	Deeplab V3+ for segmentation, PBVM for movement amplification	70	Accuracy = 93.04%	[130]
Calves	Deep Learning	Breathing Pattern	Images	Mask R-CNN	5	R^2^ = 0.91	[132]
Dairy Cows	Deep Learning	Respiration Rate	Video	Optical Flow	50	Accuracy = 92.40%	[128]
Dairy Cows	Deep Learning	Respiratory Rate	Infrared Thermography Images	YOLOv8	5	Accuracy = 94.58%	[133]
Calves	Deep Learning	Respiratory Behavior	Video	YOLOv5	50	Accuracy = 96.80%	[136]
Dairy Cows	Deep Learning	Respiration Rate	Video	YOLOv5 for detection/segmentation, LK optical flow analysis	219	Accuracy = 94.40%	[131]
Dairy Cows	Deep Learning	Respiration Rate	Video	End-to-End Transformer Model (VideoMAE)	6	MAE = 2.58 BPM	[137]
Dairy calves	Deep Learning + Machine Learning	Respiratory pattern	Images	Mask R-CNN + thermal signal extraction	11	Accuracy = 72.00%	[135]
Not specified	Deep Learning	Breathing Pattern Detection	Video	Optical Flow (LK), Transfer Learning, Ensemble Models	Not specified	Accuracy = 95.31%	[141]
Dairy cattle	Deep Learning + Machine Learning	Flank/abdominal movement from whole-body segmentation	Video	Segment Anything Model (SAM) (segmentation) + area/contour-based feature extraction (abdomen-focused)	Not specified	MAE = 2.11 BPM	[138]
Dairy cattle	Machine Learning + Deep Learning	Flank/abdominal region pixel intensity variation	Video	Image analysis + Fast Fourier Transform (FFT)	30	R^2^ = 0.75–0.80	[142]
Dairy cattle	Deep Learning + Machine Learning	Nostril region temperature fluctuation)	Video	YOLOv8-Pose+ Random Forest + dual-nostril curve fusion strategy	176	Accuracy = 96.30%	[134]
Dairy cattle	Deep Learning	Flank/abdomen movement, OSPABB region best)	Video	R2Plus1D18 + MViTv2 (Vision Transformer)	84	R^2^ = 0.79–0.81	[139]

**Table 7:** Summary of cattle behavior recognition method using computer vision.

Cattle Type	AI Subset	Measurement	Input	Model Name	Number of Animals	Model Performance	Reference
Dairy Cows	Deep Learning	Head, Back, Legs Detection	Video	FLYOLOv3 with FilterLayer	1000	Accuracy = 99.18%	[149]
Dairy Cows	Deep Learning	Basic motion behaviors	Video	Rexnet 3D network	Not specified	Accuracy = 95.00%	[147]
Dairy Cows	Deep Learning	Daily Behavior Recognition	Image	ResNet50	90	Accuracy = 85.00%	[152]
Dairy Cows	Deep Learning	Tracking and Detection	Video	YOLO v7	Not specified	Accuracy = 97.30%	[154]
Beef Cattle	Deep Learning	Nine-axis IMU (acceleration, angular velocity, magnetic field)	Transformed IMU data into images	EdgeNeXt	12	Accuracy = 95.85%	[150]
Beef Cattle	Deep Learning	Behavior Detection and Tracking	Video	YOLOv8	7	Accuracy = 93.60%	[143]
Dairy Cows	Deep Learning	Behavior Recognition and Multi-Object Tracking	Video	YOLOv8	Not specified	Accuracy = 91.70%	[144]
Dairy cattle	Deep Learning + Machine Learning	Whole body appearance + movement tracking	Video	YOLOv8 + VGG16 (feature extraction)	~1263	Accuracy = 95.00%	[145]
Dairy cattle	Deep Learning	Whole body shape and appearance across growth stages	Video	Siamese Neural Network (SNN) with ResNet-50 backbone	106	Accuracy = 94.60%	[153]
Beef/dairy cattle	Deep Learning + Machine Learning (hybrid)	Whole-body movement trajectory / spatial position over time	Video	YOLOv8/Detectron2 (detection)	20	Accuracy = 98.70%	[148]
Dairy cattle	Deep Learning	Feeding behavior; whole body position relative to feeder	Video	YOLOv8n	19	Accuracy = 88.00%	[151]
Dairy cattle	Deep Learning	Face region: nose, eyes, mouth landmarks	Video	CFR-YOLO (YOLOv7)	Not explicitly specified	Accuracy = 98.46%	[155]
Dairy cattle	Deep Learning	Behavior support / identification	Video	YOLOv9/YOLOv10/YOLOv11/YOLOv12	90	Accuracy = 92.50%	[156]
Dairy cattle	Deep Learning + Machine Learning	Behavior support / identification (ear tag region)	Video	YOLOv5s (ear tag detection) + NVIDIA DeepStream tracking	550	Accuracy = 90.10%	[157]
Dairy calves	Deep Learning	Whole body posture: inside hutch, outside standing, outside lying	Image	YOLOv3 and YOLOv3-tiny (object detection)	12	Accuracy = 97.30%	[158]
Beef/dairy cattle	Deep Learning	Whole body movement and spatial position relative to virtual boundary	Video	YOLOv11 (object detection) + DeepSORT (multi-object tracking)	Not explicitly specified	Precision 0.89–0.99, Recall 0.85–0.96	[159]
Dairy/beef cattle	Deep Learning	Face region: eyes, nose, facial structure	Image	ILS-pyramid (image enhancement) + EMA-YOLOv8 (face detection) + IGAM-iResNet	1957	Accuracy = 99.85%	[160]
Dairy cattle	Deep Learning	Whole body posture: standing vs. lying	Video	YOLOv5	100	Accuracy = 96–97%	[161]
Dairy cattle (pasture environment)	Deep Learning + Machine Learning (hybrid)	Whole body position + head location via UWB tag	Images	YOLOv5s (object detection) + UWB localization + BP Neural Network (error correction)	1	-	[162]
Beef cattle	Deep Learning	Whole body activity + spatial position	Video	YOLOv5 (behavior detection and classification) + YOLOv8 Pose (ground point estimation)	17	mAP ≈ 99.2%	[163]
Dairy cattle	Deep Learning	Whole body activity: eating, drinking, resting, standing	Images	YOLOv7 (behavior detection) + Multitask Contrastive Network (MTCN, Siamese-based) for individual identification	21	Accuracy = 83.60%	[164]
Dairy cattle	Deep Learning + Machine Learning	Behavior support / physiological pattern (eye region temperature)	Thermal infrared images	YOLOv8 (head detection) + OH-YOLO (eye region segmentation)	28	R^2^ = 0.852	[165]
Dairy cattle	Deep Learning	Whole body posture and activity: standing, lying, eating, drinking	Video	Res-DenseYOLO (improved YOLOv5 with DenseNet + CoordAtt attention + multi-detection heads + SIoU loss)	90	mAP = 4.5%	[146]
Dairy cattle	Deep Learning	Whole body movement and spatial tracking	Video	YOLO11m (object detection) + SAMURAI (segmentation, based on SAM2.1)	10–14	Accuracy = 98.8–99.9%	[166]

## Data Availability

This article is a review of published literature. No new data were created or analyzed in this study. All relevant information is contained within the manuscript and its cited references.

## Abbreviations

The following abbreviations are used in this manuscript:

PLF	Precision livestock farming
BCS	Body Condition Scores
GMM	Gaussian Mixture Model
RFB_Net_SSD	Receptive Field Block Network with Single Shot MultiBox Detector
Mask R-CNN	Mask Regional-Convolutional Neural Network
Faster R-CNN	Faster Region-Based Convolutional Neural Network
YOLO	You Only Look Once
SVM	Support Vector Machine
LSTM	Long Short-Term Memory
DenseNet	Densely Connected Convolutional Network
CART	Classification and Regression Trees
CatBoost	Categorical Boosting
SEN	Spatiotemporal Energy Network
SOLO	Segmenting Objects by Locations
AdaBoost	Adaptive Boosting
MLP	Multilayer Perceptron
LiDAR	Light Detection and Ranging
PointNet++	Deep Hierarchical Feature Learning on Point Sets in a Metric Space
DT	Decision Trees
GB	Gradient Boosting
KNN	K-nearest neighbors
RF	Random Forests
ResNet	Residual Network
SE Mechanism	Squeeze-and-Excitation Mechanism
BP Neural Network	Backpropagation Neural Network
VGG	Visual Geometry Group
ANN	Artificial Neural Network
SIFT	Scale-Invariant Feature Transform
DeepSORT	Deep Simple Online and Realtime Tracking
ViT	Vision Transformer
CNNs	Convolutional neural networks
DRN	Dense Residual Network
PBVM	Phase-based video magnification
LK	Lucas-Kanade
KLT	Kanade–Lucas–Tomasi
DSConv	Dynamic Snake Convolution
DyConv	Dynamic convolution
BiFormer	Vision Transformer with Bi-Level Routing Attention
C2f	Cross Stage Partial with Two Convolutions
iRMB	Inverted Residual Mobile Block
ConvBNSwish	Convolution, Batch Normalization, and the Swish activation function
SENet	Squeeze-and-Excitation Network
SGD	Stochastic gradient descent
EdgeNeXt	Efficiently Amalgamated CNN-Transformer Architecture for Mobile Vision Applications
SAM	Segment anything model

## Appendix A

**Table A1:** Search strategies and records retrieved.

Database	Search String	Records Retrieved
ScienceDirect	(cattle OR cow) AND (monitoring OR detection OR recognition OR tracking) AND (computer vision OR deep learning OR machine learning)	96
Scopus	TITLE-ABS-KEY((dairy cow OR beef cattle OR calf OR cattle OR cow OR heifer) AND (monitoring OR detection OR recognition OR tracking) AND (computer vision OR deep learning OR machine learning))	29
Web of Science	TS=((dairy cow OR beef cattle OR calf OR cattle OR cow OR heifer) AND (monitoring OR detection OR recognition OR tracking) AND (computer vision OR deep learning OR machine learning))	112
Wiley Online Library	(dairy cow OR beef cattle OR calf OR cattle OR cow OR heifer) AND (monitoring OR detection OR recognition OR tracking) AND (computer vision OR deep learning OR machine learning)	30
SpringerLink	(dairy cow OR beef cattle OR calf OR cattle OR cow OR heifer) AND (monitoring OR detection OR recognition OR tracking) AND (computer vision OR deep learning OR machine learning)	45
Google Scholar	Search 1: (dairy cow OR beef cattle OR cattle OR cow) AND “computer vision”; Search 2: (calf OR heifer) AND (deep learning OR machine learning) AND (monitoring OR detection OR tracking)	165
Total		477
